# Prevalence of high-risky behaviors in transmission of HIV among high school and college student MSM in China: a meta-analysis

**DOI:** 10.1186/s12889-015-2614-4

**Published:** 2015-12-21

**Authors:** Zhongrong Yang, Zhaohui Huang, Zhengquan Dong, Sichao Zhang, Jiankang Han, Meihua Jin

**Affiliations:** Huzhou Center for Disease Control and Prevention, Huzhou, 313000 Zhejiang Province China; Anhui Provincial Family Planning Institute of Science and Technology, Hefei, 230000 Anhui Province China

**Keywords:** Epidemiology, HIV, Men who have sex with men (MSM), AIDS, Meta-analysis

## Abstract

**Background:**

The aim of this study was to investigate the prevalence of high-risky behaviors, such as unprotected anal intercourse (UAI) in the last 6 months, condom using in their last homosexual anal intercourse, No. of male partners in the last 6 months among high school and college male students who have sex with men (MSM) in China.

**Methods:**

The relevant trials were retrieved up to June 2015 from several public databases, and a meta-analysis was conducted according to the published studies. The estimated rate and its 95 % confidence intervals (*CI*) of the relevant indexes among high school and college student MSM were collected and calculated using a fixed-effects model (the Mantel-Haenszel method) or a random-effects model (the DerSimonian and Laird method) when appropriate.

**Results:**

A total of 15 studies (18 research data), including 3297 student MSM, were performed in this meta-analysis. The overall results showed that the rate of student MSM who reported having had UAI in the last 6 months was 65.2 % (95 % *CI* = 60.2 % to 70.1 %), the prevalence of student MSM having more than one male partner in the last 6 months was 58.2 % (95 % *CI* = 51.1 % to 65.4 %), the rate of student MSM who reported using a condom in their last homosexual anal intercourse experience was 57.5 % (95 % *CI* = 49.8 % to 65.1 %), the prevalence of student MSM who were infected with HIV was 3.8 % (95 % *CI* = 2.5 % to 5.1 %), and the rate of student MSM who were infected with syphilis was 4.6 % (95 % *CI* = 3.8 % to 5.4 %).

**Conclusions:**

There are high UAI prevalence and low condom using rate in the last homosexual anal intercourse experience among high school and college student MSM in China, and corresponding control measures for this group and more effective health education of student MSM are required to prevent HIV or sexually transmitted diseases from spreading to the general population.

## Background

In recent years, HIV transmission has remained a worldwide public health concern [1–3]. An effective HIV vaccine is elusive [4], but Pre-Exposure Prophylaxis (PrEP), that means antiretroviral chemoprophylaxis before HIV exposure is a encouraging way in preventing HIV seroconversion [5, 6]. The population of men who have sex with men (MSM) via unprotected anal intercourse (UAI) or have multiple sexual partners are a major public health concern as well, particularly in the western world and some developing countries including China. MSM carry a serious burden of new HIV infections [7, 8], and are at high risk for HIV acquisition and transmission [9–13]. HIV/AIDS affects an individual physically, mentally, socially, and financially [14]. A person infected with HIV experiences systemic T-cell destruction and cell-mediated immunity reduction that lead to a wide range of opportunistic infections and cancers [15].

An increasing numbers of studies show that MSM and UAI are becoming common in China and that MSM are a hidden, however, emerging population susceptible to HIV infection [16–19]. Although MSM are a key population globally for the HIV epidemic, the HIV epidemiological data of the related indexes (such as AIDS-related knowledge, the gender of the first sexual partner, having more than one male partner in the last 6 months) in high school and college student MSM in China are sparse. We conducted this meta-analysis to investigate the prevalence of high-risky behaviors among high school and college male students who have sex with men in China, the following key indexes were studied: the AIDS-related knowledge rate, whether the gender of the first sexual partner is male, the prevalence of having more than one male partner in the last 6 months, the rate of having homosexual anal intercourse in the last 6 months, the prevalence of using a condom during the last homosexual anal intercourse, the rate of having UAI in the last 6 months, and HIV infection and syphilis infection among high school and college student MSM in China.

## Methods

### Data sources, search strategy and selection criteria

We retrieved the relevant trials up to June 2015 from several public databases, including PubMed, Springer, the Web of Science, Elsevier Science Direct, the Cochrane Library, Google scholar, China National Knowledge Infrastructure (CNKI) and the Chinese Wanfang database. The key search words used were (“HIV” or “human immunodeficiency virus” or “AIDS” or “Acquired Immune Deficiency Syndrome”) and (“UAI” or “unprotected anal intercourse”) and (“gay” or “homosexual” or “men who have sex with men” or “MSM”) and (“research” or “survey” or “study” or “trial”). The references from the retrieved papers were checked for additional studies. The selection criteria are as follows: (1) the reports of qualitative studies were collected from full-published papers and not from meeting or conference abstracts; (2) the studies reported data for UAI among high school and college student MSM in China; and (3) the studies recruited the effect size of two or more related indexes, including AIDS-related knowledge, the gender of the first sexual partner, having more than one male partners in the last 6 months, having homosexual anal intercourse in the last 6 months, condom use during the last homosexual anal intercourse, UAI in the last 6 months, and HIV infection and syphilis infection with the percentage (%) and its 95 % confidence interval (95 % *CI*). We excluded studies that were reviews or reports, duplicated studies and records.

### Extraction of the data

The data items included the study details (e.g., the first author’s name, the research year of the study, the location of the participants, the sampling methods of studies, and the HIV infection and syphilis infection status.) and the characteristics of the participants (e.g., the age and sample size). Two investigators (YZ and HZ) extracted the data independently using the standard protocol, and the third investigator (JM) reviewed their results regarding the studies. We recorded the first author’s name, research year of the study, location of the study, sample size, the participants’ age, sampling methods, whether the participants reported a college or higher educational level, rate of self-reported sexual orientation as gay, HIV infection and syphilis infection among high school and college students in China.

### Quality assessment

The quality of the included studies was evaluated according to the criteria of cross-sectional/prevalence study quality recommended by the Agency for Healthcare Research and Quality (AHRQ). This criterion contains 11 items with a “Yes/No/Unclear” response option: “Yes” was scored “1”, and “No” or “Unclear” was scored “0”. The articles were scored as follows: low quality (0–3), moderate quality (4–7), and high quality (8–11) [20].

### Meta-analysis methods

The effect size (*ES*) which is the overall merged percentage (%) and its 95 % confidence interval (95 % *CI*), was estimated for each study. The overall or pooled estimate of the merged percentage was obtained using the Mantel–Haenszel method in the fixed effect model [21] or using the DerSimonian and Laid method in the random effect model [22]. We assessed the within- and between-study variation or heterogeneity by testing the Cochran’s Q-statistic [23]. Additionally, we also quantified the effect of heterogeneity using *I*^2^ = 100 % × (*Q* − df)/*Q* [24]. A significant *Q*-statistic (*P* < 0.10) or *I*^2^-statistic (*I*^2^ > 50 %) indicated heterogeneity across the studies, and then the random effects model was used for the meta-analysis. Otherwise, the fixed effects model was used.

### Evaluation of publication bias

We measured the asymmetry of the funnel plot by using Egger’s linear regression, which assessed funnel plot asymmetry using the natural logarithm scale of the *ES*.

This meta-analysis was performed using STATA v.11.0 (Stata Corporation, College Station, TX, USA) software.

## Results

### The characteristics of the eligible studies

**Fig. 1:**
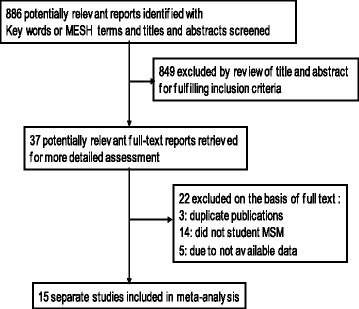
Flow diagram for selecting a study for meta-analysis

There were 886 potential relevant studies identified with the key words, titles and abstracts screened. The study selection process is shown in Fig. 1. There were 37 potentially relevant full-text reports retrieved for more detailed assessment after removing the irrelevant studies by review of title and abstract. In total, 22 of the studies were excluded (3 were duplicate publications; 14 did not report student MSM; and 5 did not provide the available data). Finally, 15 separate studies were included in this meta-analysis.

As is shown in Table 1, 15 studies (18 research data) [25–39] were included in this meta-analysis, and the characteristics of the included studies are presented. A total of 3297 student MSM were considered in the meta-analysis. The included studies were published between 2007 and 2013. The sample sizes of the studies were between 89 and 436, and the sampling method of the study was respondent-driven sampling (RDS), snowballing, MSM peer, the Internet, the voluntary counseling and testing (VCT) clinic, and non-governmental organization (NGO). Six studies [25, 26, 35–37, 39] were assessed as being of high quality, and the remainder of the studies were assessed to be of moderate quality.

**Table 1:** Characteristics of studies included in the meta-analysis

Study	Study year	Location	Sampling method	Sample size	Education	Age (Mean or Min-Max)	College or above (%)	Self-reported sexual orientation as gay (%)	HIV infection^a^(%)	Syphilis infection^b^(%)	Scores of study quality^c^
Ruan Y, 2007 [25]	2005	Beijing	The Internet, MSM peer	108	High school or below (NA) and College or above (NA)	NA	NA	NA	1.8	4.6	10
Zhang X, 2007 [26]	2005-2006	Beijing	VCT clinic	130	High school or below (NA) and College or above (NA)	NA	NA	NA	1.5	NA	10
Zhu JL,2007 [27]	2005	Hefei	RDS, MSM peer, the Internet	121	High school(25) and college students (96)	18-29	79.5	68.0	1.7	7.4	6
Chen GM, 2010 [28]	2008	Wuhu	Snowballing	129	College students	17–28	NA	71.8	NA	NA	5
Feng LG,2010 [29]	2006	Chongqing	Snowballing	210	High school or below (80) and College or above (130)	20.2	61.9	71.9	4.3	7.1	5
Feng LG,2010 [29]	2007	Chongqing	Snowballing	206	High school or below (74) and College or above (132)	20.3	64.1	81.1	3.9	3.9	5
Feng LG,2010 [29]	2008	Chongqing	Snowballing	181	High school or below (21) and College or above (160)	21.4	88.4	71.3	11.0	2.8	5
Feng LG,2010 [29]	2009	Chongqing	Snowballing	190	High school or below (39) and College or above (151)	21.4	79.5	74.2	11.1	5.8	5
Wang LX,2010 [30]	2005–2006	9 cities^b^	Snowballing	324	College students	21.6	NA	NA	0.6	NA	5
Zhou C, 2010 [31]	2008	Chongqing	Snowballing	253	High school or below (26) and College or above (227)	18–27	89.7	76.3	9.1	3.6	5
Du GY,2011 [32]	2010	Liaocheng	Snowballing	89	High school or below (NA) and College or above (NA)	17–25	NA	71.7	1.1	2.3	6
He QY,2011 [33]	2008	Chengdu	Snowballing	169	High school or below (26) and College or above (143)	13–27	84.6	71.6	8.9	5.3	5
Xi QH, 2011 [34]	2009	Nanchang	Snowballing	78	College students	20.8	100	NA	NA	NA	5
Xu JJ,2011 [35]	2008–2009	Liaoning	MSM NGO invited	436	High school(164) and college students (272)	NA	37.6	57.8	3.0	5.0	9
Zheng JD,2011 [36]	2007	Beijing	The Internet, NGO	157	College students	NA	100.0	77.7	2.5	7.0	10
Zhang L,2012 [37]	2009	Chongqing	RDS	183	High school or below (29) and College or above (154)	NA	84.2	74.9	5.5	4.4	9
Chen LF, 2013 [38]	2012	Sanya	Snowballing	128	High school or below (NA) and College or above (NA)	16–26	NA	NA	1.6	5.5	5
Wei S, 2013 [39]	2008	4 cities^a^	Snowballing and NGO	205	High school or below (NA) and College or above (NA)	16–24	NA	NA	2.9	4.9	9

^a^For most of the studies, the presence of HIV-1 antibody was initially tested by enzyme-linked immunosorbent assay (ELISA), and positive/indeterminate tests were confirmed by HIV-1/2 Western Blot (WB) assay

^b^For most of the studies that tested for syphilis, participants with serum positive for both Treponema pallidum particle assay (TPPA) and rapid plasma regain (RPR) were determined to be currently infected with syphilis

^c^Quality score is evaluated by Agency for Healthcare Research and Quality (AHRQ)

*NA* not available

### Overall effects of related indexes among student MSM

Table 2 shows the summary of the meta-analysis for the related indexes including AIDS-related knowledge, the gender of the first sexual partner, having more than one male partners in the last 6 months, having homosexual anal intercourse in the last 6 months, condom use during the last homosexual anal intercourse, UAI in the last 6 months, and HIV infection and syphilis infection among high school and college student MSM. We used the random effects model to calculate the related indexes by means of the heterogeneity test between studies except for syphilis infection (which were used the fixed effects model).

**Table 2:** Meta-analysis of the related indexes among high school and college student MSM

Overall effects	Sample size	No. of studies	Model	Estimated rate (%)	*95 % CI* (%)	Test of heterogeneity		
						*Q*	*P* value	*I* ^*2*^ (%)
AIDS-related knowledge	1754	8	Random	83.9	78.0 to 89.9	228.0	<0.01	95.6
The first sexual partner is male	1298	4	Random	85.3	82.4 to 88.3	14.0	0.03	57.1
Having more than one male partner in the last 6 months	1851	7	Random	58.2	51.1 to 65.4	89.9	<0.01	90.0
Having homosexual anal intercourse in the last 6 months	1529	7	Random	81.9	76.7 to 87.1	74.2	<0.01	87.9
Using condom during the last homosexual anal intercourse	1621	7	Random	57.5	49.8 to 65.1	94.7	<0.01	90.5
UAI in the last 6 months	2671	11	Random	65.2	60.2 to 70.1	96.61	<0.01	86.5
HIV infection	3090	13	Random	3.8	2.5 to 5.1	78.41	<0.01	80.9
Syphilis infection	2636	11	Fixed	4.6	3.8 to 5.4	11.08	0.6	0

*UAI* unprotected anal intercourse; *AI* anal intercourse

**Fig. 2:**
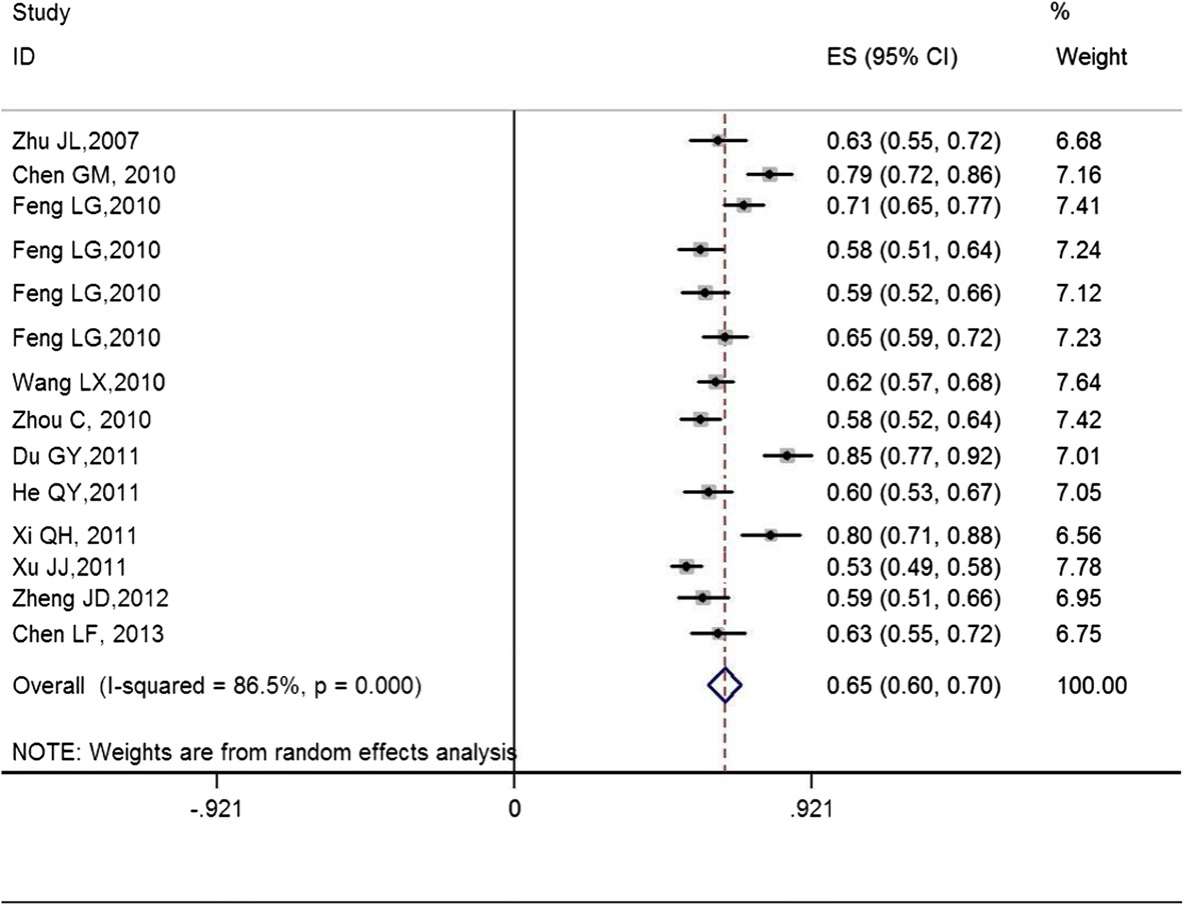
Forest plot for UAI of student MSM in the meta-analysis

The overall meta-analysis showed the following: the rate of student MSM who had AIDS-related knowledge was 83.9 % (95 % *CI* = 78.0 % to 89.9 %, *P* < 0.05); the prevalence of student MSM whose first sexual partner was male was 85.3 % (95 % *CI* = 82.4 % to 88.3 %, *P* < 0.05); the rate of student MSM who had more than one male partner in the last 6 months was 58.2 % (95 % *CI* = 51.1 % to 65.4 %, *P* < 0.05); the prevalence of student MSM who had homosexual anal intercourse in the last 6 months was 81.9 % (95 % *CI* = 76.7 % to 87.1 %, *P* < 0.05); the rate of student MSM who used a condom in the last homosexual anal intercourse was 57.5 % (95 % *CI* = 49.8 % to 65.1 %, *P* < 0.05), the prevalence of student MSM who had UAI in the last 6 months (the forest plots are shown in Fig. 2) was 65.2 % (95 % *CI* = 60.2 % to 70.1 %, *P* < 0.05); the rate of student MSM who were infected with HIV was 3.8 % (95 % *CI* = 2.5 % to 5.1 %, *P* < 0.05); and the prevalence of student MSM with syphilis was 4.6 % (95 % *CI* = 3.8 % to 5.4 %, *P* < 0.05).

### Evaluation of publication bias

**Fig. 3:**
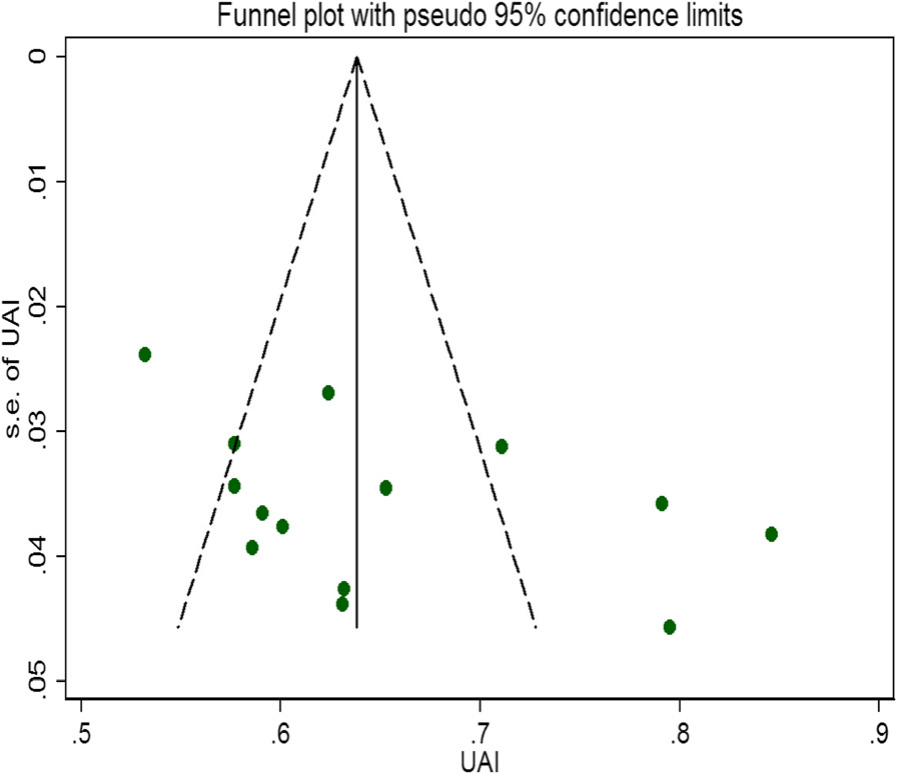
Funnel plot for UAI of student MSM in the meta-analysis

We assessed the funnel plot asymmetry using Egger’s linear regression test, which showed that there was no publication bias in UAI (*t* = 1.73, *P* = 0.11, Fig. 3).

## Discussion

The HIV prevalence among high school and college student MSM in this meta-analysis was 3.8 %, which is over sixty times higher than that in general population (0.057 %) of China [35]. This study suggests that of the high school and college student MSM in China, 83.9 % had AIDS-related knowledge, 85.3 % had a male as their first sexual partner, and 81.9 % of them had homosexual AI in the last 6 months. A public health problem that deserves attention is that sexual network of college students is centered on their school, and student MSM infected with sexually transmitted diseases or HIV could easily spread venereal diseases to his college student sex network, which in turn is likely to spread to the general population as well. For student MSM with high HIV/AIDS-related knowledge and a history of widespread unsafe sex, it is necessary to explore effective intervention measures to prevent sexually transmitted disease or HIV/AIDS in the future.

This study suggests that the knowledge and behavior among student MSM are inconsistent, showing that AIDS knowledge level is not the only factor of AIDS’ high risky behaviors; when target groups have a certain level of knowledge, we should focus on high risk behavior that affects the groups in terms of more profound social, psychological and personal factors rather than blindly raise the level of knowledge as the key point of intervention activities.

In the MSM population, students have a higher educational level and the proportion of student MSM using protective condoms is obviously higher than that of MSM, in general. Actually, student MSM could volunteer to promote HIV intervention work (such as dispense condoms and publicity material of health education in students with community activities, receive voluntary HIV testing and counseling at school), which could facilitate a reduction in the HIV infection rate in the MSM population. Regarding health initiative interventions, peer education programs using MSM should include having MSM provide free condoms and lubricants to MSM; the goal of public health programs should be to actively change behavior in MSM, to promote safe sex in MSM, and increase the knowledge of the importance of using condoms by those participating in MSM sex.

Having UAI and several sexual partners is behavior with high risks for MSM and a major risk factor for HIV infection. The condom utilization prevalence is quite high in “one night sex” with an unfamiliar sexual partner; however, inconsistent use of condoms with a boy friend (BF) or familiar sexual partner is a potential hazard of HIV or sexually transmitted diseases. Most MSM do not know whether their BFs or acquaintances have had other sexual partners and thus whether to insist on using a condom during sex.

The development of society and progress of science and technology have resulted in widespread use of the Internet. The Internet as a communication medium provides a convenient platform for MSM to find sexual partners and thus increases the likelihood of risky sexual behaviors. Most MSM look for a partner via the Internet. The Internet has increased the risk of MSM having a greater number of sexual partners. In addition, because of Chinese social and cultural factors, same-sex relationships/behavior is not typically approved of and a MSM might try to conceal his identity and activities; most MSM enter heterosexual marriages, which inevitably increases the risk of the spread of sexually transmitted diseases or HIV/AIDS to the general population.

Using the Internet to meet sexual partners is associated with an increase in HIV risk behaviors, including substance use, sex with multiple or anonymous partners, and unprotected anal sex, in diverse samples of MSM [40]. Education concerning HIV prevention has been shown to reduce or delay high-risk sexual behaviors in young MSM [41].

Educating students regarding HIV/AIDS or sexually transmitted diseases should not be a mere formality, and it should be begun at the middle school level. Health education regarding sexual disease transmission should illustrate the harm and highlight the dangers of MSM who is not using a condom when engaging in penetrative sex. Future studies should consider skills-training programs to assist MSM youth in the disclosure process and to facilitate the determination of the degree to which friends and family members could be safely disclosed to and programs to support family-based interventions [42].

This study includes some limitations. First, the heterogeneity in this study is high, which could be derived from clinical heterogeneity and statistical heterogeneity; in this study the reason for heterogeneity might be that the indicators do not have a unified definition and the samples are from different regions. In addition, because of the small number of studies included in this meta-analysis, the retrieved studies were all cross-sectional studies and we did not perform a subgroup analysis or meta-regression analysis to explore the sources of sizeable heterogeneity that might result from a time change or regional differences. A cross-sectional study might lead to potential biases that are inherent in studies; there are possible instances of bias, particularly selection bias, because of the method of identifying MSM, and, therefore, we evaluated the quality of the included studies according to the criteria of cross-sectional/prevalence study quality recommended by the Agency for Healthcare Research and Quality (AHRQ). Meanwhile, the ground for combining high school students and college students was not well justified. As high school student and college students in China are heterogeneous in terms of social experience, sexual network complexity, and HIV awareness/perception/knowledge, etc. It may not be suitable and would result in bias to combine and generate a pooled effect size based on college or high school student as a single population.

## Conclusions

This study suggests that high school and college student MSM in China have a high UAI rate, and corresponding and incisive control measures for this population as well as more effective health education of student MSM is required to prevent HIV or other sexually transmitted diseases from spreading to the general population. The first and most important objective of these measures would be to reduce the HIV prevalence by treating MSM patients with Highly Active Anti-Retroviral Therapy (HAART).

## Abbreviations

HIV: Human immunodeficiency virus
MSM: Men who have sex with men
UAI: Unprotected anal intercourse
CNKI: China National Knowledge Infrastructure
AHRQ: Agency for Healthcare Research and Quality
VCT: The voluntary counseling and testing
